# Supplementary material for: An hourglass circuit motif transforms a motor program via subcellularly localized muscle calcium signaling and contraction
Source: eLife. 2021 Jul 2;10:e59341. doi: 10.7554/eLife.59341 (PMC8331187; doi:10.7554/eLife.59341)
Supplement: Figure 4—source data 1. [file elife-59341-fig4-data1.zip › Figure 4/Fig 4D,E - M1 genetic ablation/matlab analysis - M1 genetic ablation.docx]

**CAUTION READ ME FIRST**

Before working with this data, be informed that Eugene and I scored this dataset twice – I scored it once counting procorpus pumps, spits, and feeding pumps. He went back and then later scored it for grinder pumps. Thus, there are TWO DIFFERENT “data_” files. Make sure you use the right ones!

After running customBatchBehavior on nEx19p3_arrayMinus_grinderPumps data (I already did the arrayPlus condition).

1.a) **behavior:**

behavior_nEx2905_arrayMinus_20181203_grinderPumps=[

nEx19p3_arrayMinus_01_20181203_grinderPumps,

nEx19p3_arrayMinus_02_20181203_grinderPumps,

nEx19p3_arrayMinus_03_20181203_grinderPumps,

nEx19p3_arrayMinus_04_20181203_grinderPumps,

nEx19p3_arrayMinus_05_20181203_grinderPumps]

1.b)

behavior_nEx2905_arrayMinus_20181204_grinderPumps=[

nEx19p3_arrayMinus_01_20181204_grinderPumps,

nEx19p3_arrayMinus_02_20181204_grinderPumps,

nEx19p3_arrayMinus_03_20181204_grinderPumps,

nEx19p3_arrayMinus_04_20181204_grinderPumps,

nEx19p3_arrayMinus_05_20181204_grinderPumps]

1.c)

behavior_nEx2905_arrayMinus_toDate_grinderPumps=[

behavior_nEx2905_arrayMinus_20181203_grinderPumps,

behavior_nEx2905_arrayMinus_20181204_grinderPumps]

1.d)

missingCompiled_nEx2905_arrayMinus_toDate_grinderPumps=[

missingCompiled_nEx2905_arrayMinus_20181203_grinderPumps,

missingCompiled_nEx2905_arrayMinus_20181204_grinderPumps]

*Saved above as* ***data_nEx2905_arrayMinus_toDate_grinderPumps****:*

Plotting graphs:

1. To plot overall grinder rate against spitting rate for the array minus animals:

plotMsDataFromVideo('Array Minus, grinder pumps v spits',behavior_nEx2905_arrayMinus_toDate_grinderPumps,missingCompiled_nEx2905_arrayMinus_toDate_grinderPumps,behavior_nEx19p3_arrayMinus_toDate(3,:),missingCompiled_nEx19p3_arrayMinus_toDate,0,30,1,0,0)

*saved as* ***PLOT_nEx2905_arrayMinus_toDate_grinder_v_spits***

Annoyingly, because we scored the grinder pumps and spits in different sessions (I forgot to count the grinder when I did spits), the spitting rate is higher than the grinder pumping rate in this graph.

1. Plot overall grinder rate against spits in arrayPlus animals:

plotMsDataFromVideo('Array Plus, M1 minus, grinder pumps v spits',behavior_nEx2905_arrayPlus_M1minus_toDate_grinderPumps,missingCompiled_nEx2905_arrayPlus_M1minus_toDate_grinderPumps,behavior_nEx19p3_arrayPlus_M1minus_toDate (3,:),missingCompiled_nEx19p3_arrayPlus_M1minus_toDate,0,30,1,0,0)

*saved as* ***PLOT_nEx2905_arrayPlus_M1minus_toDate_grinder_v_spits***

1. Plot overall grinder rate against procorpus in arrayMinus animals:

plotMsDataFromVideo('Array Minus, grinder pumps v spits',behavior_nEx2905_arrayMinus_toDate_grinderPumps,missingCompiled_nEx2905_arrayMinus_toDate_grinderPumps,behavior_nEx19p3_arrayMinus_toDate (1,:),missingCompiled_nEx19p3_arrayMinus_toDate,0,30,1,0,0)

1. Plot overall grinder rate against procorpus in arrayPlus animals:

plotMsDataFromVideo('Array Plus, M1 minus, grinder pumps v spits',behavior_nEx2905_arrayPlus_M1minus_toDate_grinderPumps,missingCompiled_nEx2905_arrayPlus_M1minus_toDate_grinderPumps,behavior_nEx19p3_arrayPlus_M1minus_toDate (1,:),missingCompiled_nEx19p3_arrayPlus_M1minus_toDate,0,30,1,0,0)

1. Plot overall grinder rate of array minus animals against the overall grinder rate of arrayPlus animals:

plotMsDataFromVideo('Grinder pumps arrayPlus v arrayMinus',behavior_nEx2905_arrayMinus_toDate_grinderPumps,missingCompiled_nEx2905_arrayMinus_toDate_grinderPumps,behavior_nEx2905_arrayPlus_M1minus_toDate_grinderPumps,missingCompiled_nEx2905_arrayPlus_M1minus_toDate_grinderPumps,0,30,1,0,0)

1. array minus: pumps v spits

plotMsDataFromVideo('Array Minus, grinder pumps v spits',behavior_nEx2905_arrayMinus_toDate_grinderPumps,missingCompiled_nEx2905_arrayMinus_toDate_grinderPumps,behavior_nEx19p3_arrayMinus_toDate(3,:),missingCompiled_nEx19p3_arrayMinus_toDate,0,30,1,0,1)

now array positive, pumps v spits:

plotMsDataFromVideo('Array Plus, M1 minus, grinder pumps v spits',behavior_nEx2905_arrayPlus_M1minus_toDate_grinderPumps,missingCompiled_nEx2905_arrayPlus_M1minus_toDate_grinderPumps,behavior_nEx19p3_arrayPlus_M1minus_toDate (3,:),missingCompiled_nEx19p3_arrayPlus_M1minus_toDate,0,30,1,0,1)

Now arrayMinus v arrayPlus, comparing grinder rates:

plotMsDataFromVideo('Grinder pumps arrayPlus v arrayMinus',behavior_nEx2905_arrayMinus_toDate_grinderPumps,missingCompiled_nEx2905_arrayMinus_toDate_grinderPumps,behavior_nEx2905_arrayPlus_M1minus_toDate_grinderPumps,missingCompiled_nEx2905_arrayPlus_M1minus_toDate_grinderPumps,0,30,1,0,1)

Now plot procorpus against grinder in arrayPlus animals:

plotMsDataFromVideo('Array Plus, M1 minus, grinder pumps v spits',behavior_nEx2905_arrayPlus_M1minus_toDate_grinderPumps,missingCompiled_nEx2905_arrayPlus_M1minus_toDate_grinderPumps,behavior_nEx19p3_arrayPlus_M1minus_toDate (1,:),missingCompiled_nEx19p3_arrayPlus_M1minus_toDate,0,30,1,0,1)

Making plots for thesis figure 2.1:

I’m making the plots again using the newest for-publication version of the graphs, just copying the code from above.

1. To plot overall grinder rate against spitting rate for the array minus animals:

plotMsDataFromVideoSpits('Array Minus, grinder pumps v spits',behavior_nEx2905_arrayMinus_toDate_grinderPumps,missingCompiled_nEx2905_arrayMinus_toDate_grinderPumps,behavior_nEx19p3_arrayMinus_toDate(3,:),missingCompiled_nEx19p3_arrayMinus_toDate,0,30,1,0,1)

*saved as* ***PLOTpub_nEx2905_arrayMinus_toDate_grinder_v_spits***

1. Plot overall grinder rate against spits in arrayPlus animals:

plotMsDataFromVideoSpits('Array Plus, M1 minus, grinder pumps v spits',behavior_nEx2905_arrayPlus_M1minus_toDate_grinderPumps,missingCompiled_nEx2905_arrayPlus_M1minus_toDate_grinderPumps,behavior_nEx19p3_arrayPlus_M1minus_toDate (3,:),missingCompiled_nEx19p3_arrayPlus_M1minus_toDate,0,30,1,0,1)

*saved as* ***PLOTpub_nEx2905_arrayPlus_M1minus_toDate_grinder_v_spits***

1. Plot overall grinder rate of array minus animals against the overall grinder rate of arrayPlus animals:

plotMsDataFromVideoSpits('Grinder pumps arrayPlus v arrayMinus',behavior_nEx2905_arrayMinus_toDate_grinderPumps,missingCompiled_nEx2905_arrayMinus_toDate_grinderPumps,behavior_nEx2905_arrayPlus_M1minus_toDate_grinderPumps,missingCompiled_nEx2905_arrayPlus_M1minus_toDate_grinderPumps,0,30,1,0,1)

*Save as* ***PLOTpub_grinderPumps_arrayMinus_v_arrayPlus***

1. Plot overall grinder rate against procorpus in arrayPlus animals:

plotMsDataFromVideo('Array Plus, M1 minus, grinder pumps v spits',behavior_nEx2905_arrayPlus_M1minus_toDate_grinderPumps,missingCompiled_nEx2905_arrayPlus_M1minus_toDate_grinderPumps,behavior_nEx19p3_arrayPlus_M1minus_toDate (1,:),missingCompiled_nEx19p3_arrayPlus_M1minus_toDate,0,30,1,0,0)

save as: **PLOTpub_arrayPlus_grinder_v_procorpus**
